# Ecuadorian Banana Farms Should Consider Organic Banana with Low Price Risks in Their Land-Use Portfolios

**DOI:** 10.1371/journal.pone.0120384

**Published:** 2015-03-23

**Authors:** Luz Maria Castro, Baltazar Calvas, Thomas Knoke

**Affiliations:** 1 Institute of Forest Management, TUM School of Life Sciences Weihenstephan, Department of Ecology and Ecosystem Management, Technische Universität München (TUM), Freising, Germany; 2 Institute of Silviculture, TUM School of Life Sciences Weihenstephan, Department of Ecology and Ecosystem Management, Technische Universität München (TUM), Freising, Germany; 3 Departamento de Economía, Universidad Técnica Particular de Loja, Loja, Ecuador; United States Department of Agriculture, UNITED STATES

## Abstract

Organic farming is a more environmentally friendly form of land use than conventional agriculture. However, recent studies point out production tradeoffs that often prevent the adoption of such practices by farmers. Our study shows with the example of organic banana production in Ecuador that economic tradeoffs depend much on the approach of the analysis. We test, if organic banana should be included in economic land-use portfolios, which indicate how much of the land is provided for which type of land-use. We use time series data for productivity and prices over 30 years to compute the economic return (as annualized net present value) and its volatility (with standard deviation as risk measure) for eight crops to derive land-use portfolios for different levels of risk, which maximize economic return. We find that organic banana is included in land-use portfolios for almost every level of accepted risk with proportions from 1% to maximally 32%, even if the same high uncertainty as for conventional banana is simulated for organic banana. A more realistic, lower simulated price risk increased the proportion of organic banana substantially to up to 57% and increased annual economic returns by up to US$ 187 per ha. Under an assumed integration of both markets, for organic and conventional banana, simulated by an increased coefficient of correlation of economic return from organic and conventional banana (ρ up to +0.7), organic banana holds significant portions in the land-use portfolios tested only, if a low price risk of organic banana is considered. We conclude that uncertainty is a key issue for the adoption of organic banana. As historic data support a low price risk for organic banana compared to conventional banana, Ecuadorian farmers should consider organic banana as an advantageous land-use option in their land-use portfolios.

## Introduction

The intensification of agricultural systems has resulted in a substantial increase in the amount of food produced in the last decades through the application of technologies such as high-yielding crop varieties, chemical fertilizers and pesticides, irrigation and mechanization [1]. Reaching current levels of food production would hardly have been possible without the use of these technologies [2]. Nonetheless, poorly managed intensification can ultimately lead to a drop in soil fertility, pollution of ground water, increased release of greenhouse gases and overall losses in biodiversity [1, 3–6]. Such detrimental impacts on the environment and on ecosystem services highlight the need for more sustainable methods of producing food [7].

In practice, however, the adoption of sound practices, such as organic farming, is still limited, due to the economic attractiveness of conventional agriculture and government policies that continue to encourage the use of synthetic inputs [1]. In general, little is known about the economic performance of sustainable land-use practices [8], for example, organic farming. Consequently, a full accounting of both the costs and the benefits of sustainable agriculture should form the basis for policy, ethics and action [7, 9]. Indeed, assessing the ecological and economic tradeoffs between organic and conventional farming, and identifying the economic perspectives from which the adoption of organic farming could be advantageous forms a major challenge.

Evidence confirms that organic farming delivers lower yields than conventional farming [2, 10–13]; nonetheless, a positive aspect of producing organically is the meaningful reduction of external inputs such as fertilizers, energy and pesticides due to enhanced soil fertility and higher biodiversity [10]. The fact that organic systems may require 35% more labor than conventional does not make organic agriculture necessarily more expensive than conventional as reduced costs of fertilizers and pesticides represent an important component on overall costs [14]. In addition, the extra costs generated by adopting organic standards are supposed to be more than offset by the price premium that consumers pay when purchasing bananas with a sustainable agriculture label [15].

A reduction in yield for instance, does not imply that organic farming might not be attractive at all for farmers, because organic and conventional products are sold on different markets [16]. The prices for organic and conventional products may thus show merely small correlation, and price volatility may be lower for organic products [17]. In many countries, price premiums for organic products appear to be non-declining over time (e.g. for pineapples) [18]. Additionally, net returns in conventional systems have been reported to be more variable and thus more risky than in organic corn-soybean systems [14]. Moreover, the volatility of crop yields may differ between organic and conventional production. Microbial biomass and activity as well as soil organic carbon are almost always significantly higher in soils of the organic farming systems than in those of the conventional system and microbial communities are more active under the organic system. In the organic soils, microbial activity is positively correlated with soil fertility [19]. If organic farming would also achieve reduced volatility of market prices, this would suggest that organic farming systems would be a well suited option to diversify conventional land-use systems. However, to the best of our knowledge there are hardly any studies which have tested whether organic farming systems are suited as valuable components to diversify conventional land-use portfolios.

Our study will focus on banana production in Ecuador and we intend to test the following hypothesis:

H: “The inclusion of organic banana into efficient economic land-use portfolios in Ecuador is driven by the uncertainty of their economic return”

A land-use portfolio is named “efficient,” if there is no other land-use portfolio with a higher economic return for the given level of economic risk. We test the impact of the volatility of economic returns for organic banana and the influence of the correlation between economic returns from organic and conventional banana on the inclusion or exclusion of organic banana into or from the optimal land-use portfolios.

## The approach followed: Land allocation based on economic return and risk

Farmers’ decisions about how best to use their land are driven by the goal of improving their own well-being. Well-being is defined across many dimensions, including income, security of livelihood, and health [16]. Decisions about land use are influenced by the relative potential economic return or benefit of each activity, which, in turn, depends on the available technology and prevailing market and environmental conditions [20].

In general, it is reasonable to expect that farmers will choose productive activities that maximize their well-being, given the resources and opportunities available to them. However, as farmers are typically regarded as risk-averse, strategies to reduce the uncertainties inherent to agricultural production may provide beneficial effects [21, 22]. Farmers will, consequently, not only seek high average, but also low standard deviation (SD) of discounted future net revenues. Risk-averse farmers may achieve high levels of risk reduction by mixing two or more land-use options whose financial yields fluctuate independently from one another (with low correlations) [23]. In other words, in periods when returns from one asset drop, another one may generate unexpectedly high returns, thus, moderating the effects of economic booms and busts [22, 24].

The level of land-use diversification may range in intensity from intermingled cropping (e.g. agroforestry) to landscape-level approaches [1, 25]. Diversification at the landscape level consists of producing crops in separated parcels that are relatively small in size but still large enough to permit agricultural intensification (e.g. mechanization) [25]. A well-recognized method for finding the optimal diversification strategy is the Portfolio Theory [26]. This theory has been used, for instance, to further develop Thünen’s [27] economic land-use theory using a portfolio-theoretic reformulation [28]. Our paper builds on the portfolio-theoretic enhanced Thünen approach by modeling farmers’ options for balancing economic return and risk. It may be used, on the one hand, to find an appropriate land allocation to various land-use practices on new banana farmland. FAO Statistics tell us that in Ecuador, from 1980 to 2012 the area of banana farms increased by 4400 ha per year. On the other hand, also in existing farms the exhausted banana plants have to be replaced all five to ten years making it necessary to renew the investment. This gives an opportunity to alter the existing land distribution to land-use practices. For example, assume a farm with 200 ha pure banana, where banana plants have to be replaced all ten years. In order to balance the annual work a portion of 20 ha could be renewed every year so that all banana plants are replaced once during a 10-year time span. A completely new land-use portfolio may, in this way, be created within ten years without stopping the banana investment before plant productivity reduces.

Our approach attempts, by means of the allocation of land to various land-use practices, to maximize the expected economic return (in the form of the average annualized net present value), for a given level of accepted risk, which is represented by the SD of economic return, through careful selection of the proportions of total available land area occupied (so called portfolios) by various land-use options. Those portfolios that provide the largest economic return for a given SD are termed efficient portfolios. All others are considered inefficient.

Markowitz [29] proposed his famous portfolio theory in a normative sense, as a recommendation for portfolio selection, and in a positive sense, too, as a hypothesis about investor behavior. Here, we apply Markowitz’ theory in combination with the Thünen approach in a normative sense. Thus, our model shows how land *should* be allocated to the available land-use practices to achieve the highest economic return for an accepted level of risk. This does not necessarily mean that the model output is a proper prediction of future land allocation, nor will it necessarily describe the past land allocation practices. It may just help risk averse land owners to achieve their economic objectives in a consistent way. Normative models like ours may hardly be tested empirically (see Roll’s [30] critique to the Capital Asset Pricing Model), but still can help forming comprehensible land-use scenarios and delivering valuable hints for risk-return efficient land-use strategies [28]. These kinds of models have been applied in the past in order to model decisions on land allocation to various land-use practices from an economic perspective [27] and to derive cost-effective conservation strategies [28].

Our model has a static nature, although the time structure of net revenues, such as lacking of significant positive net revenues in the first three years of organic banana, are considered for the single land-use options. However, it is investigated how land should be allocated to land-use practices, but not when this should take place, because the timing of crop conversion is much influenced by the nature of the investment. For example, when banana or cocoa plants are exhausted, they must be replaced, whereas it would not be wise to stop the investment before. The optimal allocation of land to land-use practices delivered by our model is, thus, valid in general for the future, regardless when it will be achieved. Our consideration assumes that the same land-use practices with the same economic characteristics are available in each time period. Of course, new price, cost or uncertainty levels may establish themselves in future periods, which would alter economic returns, their uncertainties and correlations. However, to predict these changes at this time would be speculative. We, thus, prefer the static approach, which still allows for computing revised allocation of land to land-use practices, when new information is available. However, we have to keep in mind the static, single period nature of our analysis, which is embedded in a many-period reality [29], where the economic coefficients may actually change from period to period. To consider this we actually recommend to revise the analysis of land-use portfolios from time to time in practical applications.

We use the classical SD as a measure for risk and uncertainty, while we do not distinguish between risk and uncertainty [31]. Of course, uncertainty would cover also the right tail of the probability distribution of possible economic returns, with actual returns being higher than expected, which is not to be considered a “risk”. It is well known that available options to react in response to the actual development of prices, costs or productivities may produce economic returns that are located on the right tail of return distributions. The options to defer, abandon, contract, expand or switch the investment [32] may increase the economic return in comparison to results delivered by the classical net present value approach [33]. This flexibility can be considered on the formal basis of the options approach to capital investments [34]. However, the consideration of multiple real options in a portfolio approach is very complex. For example, the option values for the single land-use practices are not additive [32]; we, thus, should be aware that using one option could compromise the use of other options. If we exercise the option to expand or switch to organic banana, the options to expand or switch to other practices will be limited. Also, if we wait too long with exercising, competition may have eroded the option value already [35]. In making solutions to the real options problem manageable, most of the applications of the real options approach in land management, as a result, reduce their problem perspective to consider only one investment project or the replacement of only one project by another one. For example, Yemshanov et al. recently investigated when, if at all, to convert agriculture into a bio-fuel poplar plantation and vice-versa [36]. In another study Capozza and Sick priced agricultural land with a real option to convert into urban land [37]. In contrast to these studies we are interested in the optimal structure of land-use portfolios, potentially consisting of many land-use options. We are aware that the options inherent in the single land-use practices considered can have an impact on their economic returns and risks, as studies have shown for mixed forests in comparison with pure forests [38, 39]. The structure of land-use portfolios might, thus, be altered by the options approach in theory, if option values differ greatly between the land-use practices considered. Here, the land-use practices with the greatest volatility of economic returns would have the greatest potential to bear significant option values. However, these practices may also be the riskiest and (improper) option pricing may include the possibility of greatly overestimating the value of the most uncertain projects. We will discuss possible effects of applying the options approach on the composition of land-use portfolios at the end of our paper. Indeed, it would be very challenging to adequately determine an inclusive set of relevant options (e.g., timing of inclusion, exclusions, conversion and possible re-conversion) for all the land-use practices considered simultaneously. And this without inflating the economic risk—due to the incorporation of many uncertain, partly speculative elements—beyond the level which the landowners would be willing to accept. Although theoretically attractive, real options are often considered by managers to overestimate the value of uncertain projects, encouraging decision makers to overinvest in them and to gamble in the extreme case [35].

There are also technical problems, which rather detract from the real options approach. For example, Plantinga pointed out that decisions on the optimal timber harvest under uncertain prices depend strongly on the underlying process to simulate prices [40]. Also Insley showed that applying either the Geometric Brownian Motion or mean-reverting prices had a great impact on the outcome of option values and when to best harvest (replace) existing trees [41]. Due to problems with the acquisition of appropriate data and the choice of the appropriate price/cost processes plus the very complicated modelling of interacting options in a portfolio, the actual computation of option values is still considered problematic, although the conceptual value of the approach is acknowledged [42]. Some studies, thus, consider the practical application of the real options approach critical [43, 44]. In summary, we justify our static approach as being helpful to analyze the attractiveness of organic banana, because it is more informative then studies considering organic and conventional farming as mutually exclusive land-use options [13, 45, 46] and less speculative compared to the option value approach.

According to the theory of portfolio selection the expected economic return of a portfolio with two or more assets, *R*_*p*_, is obtained by adding the expected economic returns, *r*_*i*_, weighted by their proportions, *f*_*i*_, of the single land-use options.

$$R_{p}=\sum_{i}f_{i}\cdot r_{i}$$ (1)

with *r*_*i*_ as the annualized sum of all appropriately discounted (discount factor *q = 1+d* and *d* as the discount rate) net revenues, *n*_*i*_, of land-use option, *i*, over a time period, *t*, of 30 years:

$$r_{i}=\left[\sum_{t}n_{i}\cdot q^{-t}\right]\cdot \frac{\left(q-1\right)\cdot q^{30}}{q^{30}-1}$$ (2)

We applied a discount rate, *d*, of 0.05, and thus *q* = 1.05, as this discount rate has been used in the past to assess forestry and farm strategies in the tropics [47–49]. Using a higher discount rate would of course, substantially reduce the economic returns. Equation 2 converts the net present value directly into an annuity. This is practical for the modelling, because the annuity may be compared well between the land-use practices considered and has the same unit as the net revenue per year of annual crops (for which annuity and yearly net revenue is identical).

The SD of economic returns for the portfolio, *σ*_*p*_, is quantified as follows,

$$\sigma_{p}=\sqrt{\sum_{i}\sum_{j}f_{i}\cdot f_{j}\cdot {\mathrm{cov}}_{i,j}}$$ (3)

With:

$$\sum_{i}f_{i}=1\;f_{i,j}\geq 0\;{\mathrm{var}}_{i}\text{:}={\mathrm{cov}}_{i,i}\;{\mathrm{cov}}_{i,j}=\rho_{i,j}\cdot s_{i}\cdot s_{j}$$ (4)

where *i* and *j* are the indices for the specific land-use options; *f*_*i*_ is the proportion of land occupied by a specific agricultural land-use practice in the portfolio; *s*_*i*_ is the SD of returns for land-use practice *i; ρ*_*i*,*j*_ is the coefficient of correlation between the economic returns for options *i* and *j*; var_i_ is the variance and cov_*i*,*j*_ is the covariance between the economic returns for options *i* and *j*. Using this method, the effects of diversification can be identified for different combinations of land-use options, provided that the variability of their economic return is not perfectly positive correlated (ρ≠1).

## Land-use options considered and economic modeling

### Crops selected for the land-use portfolios

The area selected for our modelling—the Babahoyo sub-basin—is located in the littoral region of Ecuador. This region is a flat floodplain cross-cut by many rivers. Alluvial soils of volcanic origin prevail, which are typically well-drained sandy clay soils with variable textures. Intensive agriculture covers 65% of the land [50]. Permanent crops common to the region consist of banana (*Musa acuminata*), sugar cane (*Sacharum officinarum*), African palm (*Elaeis guineensis*), cocoa (*Theobroma cacao*) and coffee (*Coffea arabica*). The main annual crops in the region are maize (*Zea mays*), rice (*Oryza sativa*) and soybeans (*Glycine max*) [51].

**Fig 1:**
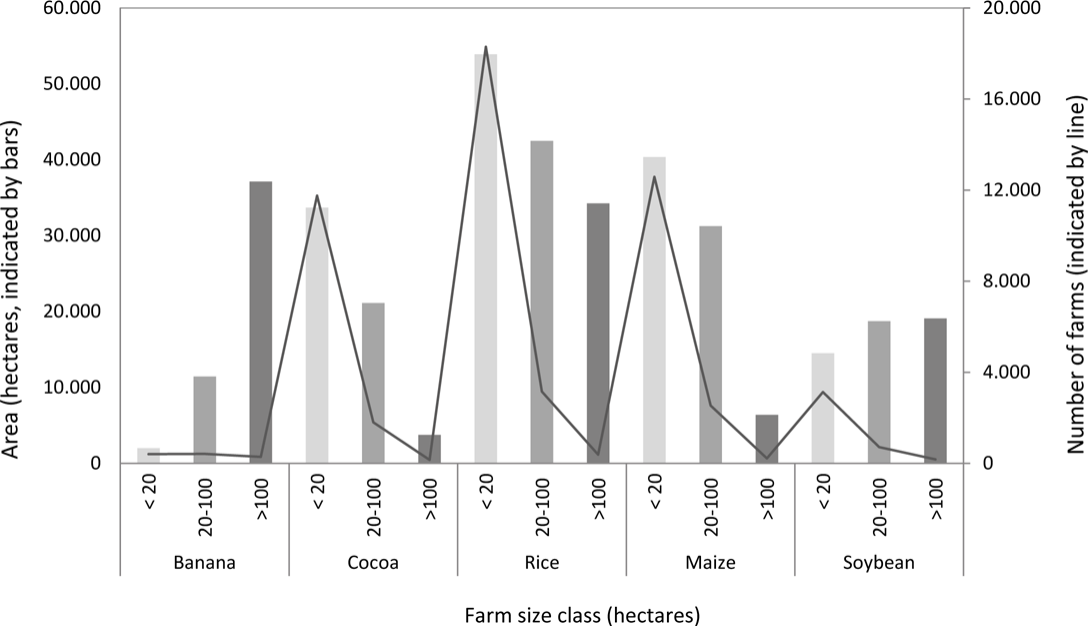
Farms in the Babahoyo sub-basin producing the land-use options modeled, arranged by size and area of production.

We modeled a typical medium-sized farm (100 hectares) across a time horizon of 30 years using the crops with the highest relevance for the region, based on information from the III Census of Agriculture and Livestock (Fig. 1). The selected land-use options were banana (conventional and organic), cocoa, rice, maize and soybean as well as two tree species.

Banana is the main export-oriented agricultural commodity in Ecuador, thus it is generally produced under very intensive management [52]. The methods and inputs used to produce banana are more intensive and expensive than every other crop used in this study (Table 1 and S1 Dataset). Due to the importance of banana to the local economy, the extent of the area currently under production and the impacts caused by the use of synthetic inputs, we considered it imperative to assess whether partial conversion to organic production might be economically attractive for local farmers. This step was shown to be feasible and may also be meaningful in terms of risk reduction, because prices for conventional and organic products are subject to different market conditions [11], thus, we may also expect positive effects of diversification. Two aspects support this assumption: organic production delivers better ecosystem services than conventional production, and the demand for organic products has risen significantly during recent years [18]. Although organic banana still represents only a small fraction of Ecuadorian banana exports (3%), the area allocated to organic banana rose nearly threefold between 2004 and 2007, from 4700 to 13800 hectares [15].

**Table 1:** Coefficients used to compute net returns of agricultural assets in the Babahoyo sub-basin.

Land-use option	Item	Coefficient	Source
Cocoa	Establishment costs	US$1606 ha^-1^	Every 15 years, own calculation
	Annual costs	US$ 353 ha^-1^	Own calculation
	Price	US$ 1694 Mg^-1^	[51]
	Yield	0.3 Mg ha^-1^	[51]
Conventional banana	Establishment costs	US$ 2835 ha^-1^	All 10 years, own calculation
	Annual costs	US$ 4745 ha^-^	Own calculation
	Price	US$292 Mg^-1^	[52]
	Yield	23 Mg ha^-1^	[52]
Organic banana	Establishment costs	US$ 3150 ha^-1^	Every 10 years, own calculation
	Annual costs	US$ 4945 ha^-1^	Own calculation
	Price	US$398 Mg^-1^	[52]
	Yield	15 Mg ha^-1^	[52]
Rice	Costs	US$ 744 ha^-1^	Own calculation
	Price	US$ 300 Mg^-1^	[51]
	Yield	3.7 Mg ha^-1^	[51]
Maize	Costs	US$ 603 ha^-1^	Own calculation
	Price	US$ 277 Mg^-1^	[49]
	Yield	3.1Mg ha^-1^	[49]
Soybean	Costs	US$ 520 ha^-1^	Own calculation
	Price	US$ 404 Mg^-1^	[51]
	Yield	1.7 Mg ha^-1^	[51]
Balsa	Establishment costs	US$ 1584 ha^-1^	All 6 years, adapted from Proforestal*
	Stand growth	45 m^3^ ha^-1^ year^-1^	
	Density	833 trees	
	Harvesting costs	US$ 600 ha^-1^	
	Revenues	US$ 6000 ha^-1^	
Laurel	Establishment costs	US$ 1554 ha^-1^	All 15 years, adapted from Proforestal*
	Stand growth	18 m^3^ ha^-1^ year^-1^	
	Density	833 trees	
	Harvesting costs	US$ 2200 ha^-1^	
	Revenues	US$ 10621 ha^-1^	
Discount rate	5%		

*Proforestal is the Office for the Promotion of Forestry in Ecuador (MAGAP)

As mentioned before, conversion from conventional to organic farming can be carried only on part of the available land [53], for example, when the existing banana plants have to be renewed anyways. This is the case according to our modelling every 10 years (see S1 Dataset). Partial conversion means that potential problems with the new production system can be better managed and buffered. For example, evidence shows that in combination with organic farming, conventional farming helps to keep levels of pests low in the organic parcels. However, the share of organic farming should not exceed certain thresholds [12].

Despite forestry is not a traditional land use in the coastal area of Ecuador, in recent years landowners have shown interest for investments in fast-growing species such as balsa (*Ochroma Pyramidale*) and laurel (*Cordia alliodora*)as a complement to agriculture. Both species are able to thrive in lands formerly dedicated to agriculture [54], which make them ideal for reforestation in abandoned or degraded land. Thus, we included these two species in our diversification modeling as a mechanism to increase the supply of timber from non-native forest species and to foster restoration of abandoned agricultural land [25, 55].

### Modeling economic performance of land uses

Price and yield statistics for each land-use option were collected from official sources at both the national and international levels (Table 1). Later, we calculated the costs and revenues for each land-use option (S1 Dataset). The costs considered included land preparation, planting, pest control, fertilization, maintenance, harvesting and infrastructure (irrigation, roads, etc.). Due to modeling constraints, we did not consider intra-annual crop rotations.

The costs considered for reforestation included those for stand establishment, protection, thinning and final harvest. Management plans detailing the intensity of interventions and the parameters of the plantations of both species are presented in Table 2. We included an estimated mortality rate of 20% of the planted tree seedlings [48, 56], and, a fluctuation in growth of 10% [48]. Returns were calculated by multiplying the number of logs harvested by the price received for raw logs.

**Table 2:** Harvest planning for balsa and laurel, adapted from Proforestal.*

Variables	Balsa		Laurel	
Age	Year 3	Year 5	Year 8	Year 15
DBH** (m)	0.20	0.35	0.20	0.35
Commercial height (m)	8	10	8	12
Form Factor	0.70	0.70	0.70	0.70
% Thinning	0.50	1.00	0.50	1.00
Volume per tree (m^3^)	0.18	0.67	0.18	0.81
Volume per ha (m^3^ ha^-1^)	58.62	224.40	58.62	269.28

*Proforestal is the Office for the Promotion of Forestry in Ecuador (MAGAP)

** Diameter at breast height

We used the prices and productivities for the period 1970–2009 published in FAOSTAT (Table 3) to model uncertainty and characteristic correlation structures between product prices and productivities. These series contain country-level data; nevertheless, we considered them to be applicable to our study area, because the region is one of the most productive areas in the country, thus the data is not overoptimistic. Prices for the period prior to the year 2000 were first converted from Sucre (Ecuador′s former currency) into US$, using annual exchange factors [57]. To adjust the historical data to the current price level, annual prices were divided by the average price of the time series, and this quotient was then multiplied by the current price for each crop. Similarly we also adjusted the data series of yields for every land-use option using the same procedure we applied to the prices.

**Table 3:** Yields and prices during the period 1970–2009 for main crops in Ecuador (obtained from FAOSTAT) [70].

Land-use options *i*										
	Cocoa		Maize		Banana		Soybean		Rice	
	Price	Yield	Price	Yield	Price	Yield	Price	Yield	Price	Yield
Year	US$	Mg	US$	Mg	US$	Mg	US$	Mg	US$	Mg
*T*	Mg^-1^	ha^-1^	Mg^-1^	ha^-1^	Mg^-1^	ha^-1^	Mg^-1^	ha^-1^	Mg^-1^	ha^-1^
1970	381.1	0.2	54.0	0.9	24.3	15.0	144.0	1.0	68.2	2.7
1971	364.5	0.3	101.2	0.7	20.2	15.2	76.4	1.1	60.1	3.5
1972	383.6	0.3	116.7	0.7	20.8	15.1	156.7	1.2	69.4	2.1
1973	791.8	0.3	162.9	0.9	26.3	15.4	187.2	1.3	84.7	2.8
1974	931.4	0.4	207.7	0.9	27.8	17.6	368.1	1.4	126.0	2.6
1975	1028.3	0.3	208.1	1.0	29.9	23.2	335.6	1.5	153.9	3.0
1976	1310.0	0.3	195.8	1.0	30.2	24.0	332.2	1.5	157.9	2.6
1977	1687.5	0.3	201.5	0.9	33.2	24.4	348.6	1.3	175.8	3.1
1978	2105.7	0.3	210.6	1.0	36.7	28.0	371.2	1.5	197.0	2.8
1979	2055.6	0.3	204.1	1.0	37.0	30.1	362.7	1.3	192.8	2.9
1980	1857.2	0.3	220.7	1.1	38.9	32.2	371.8	1.3	203.0	3.0
1981	1061.3	0.3	196.5	1.2	35.2	31.4	331.5	1.6	221.3	3.3
1982	822.5	0.4	129.7	1.5	36.3	30.7	207.4	1.8	153.0	2.9
1983	1286.8	0.2	204.7	1.1	19.9	27.7	242.1	1.4	165.9	2.9
1984	1522.9	0.3	192.5	1.3	30.1	27.7	185.2	1.7	201.1	3.1
1985	1395.5	0.5	180.5	1.6	22.6	30.2	251.5	1.8	234.8	2.7
1986	1313.3	0.3	137.0	1.0	27.7	20.7	305.0	1.9	225.1	2.5
1987	1563.4	0.2	158.9	0.9	33.6	20.0	284.9	1.8	122.7	2.8
1988	903.5	0.3	135.4	0.9	13.5	20.2	174.3	1.8	122.3	3.3
1989	891.7	0.3	134.7	1.1	115.9	19.7	266.6	1.9	181.4	3.1
1990	843.7	0.3	142.7	1.1	130.1	21.3	300.7	2.0	154.7	3.1
1991	686.2	0.3	254.2	1.1	147.3	20.9	274.3	1.9	122.0	3.0
1992	651.7	0.3	219.3	1.1	131.6	21.6	261.6	1.6	142.0	3.3
1993	725.3	0.3	455.1	1.2	126.8	21.7	263.7	1.8	139.2	3.5
1994	991.6	0.2	292.4	1.1	128.5	23.0	255.2	2.2	161.5	3.7
1995	907.7	0.2	452.8	1.1	116.5	23.7	246.1	1.1	169.2	3.3
1996	908.1	0.3	362.7	1.1	132.9	25.3	258.2	1.3	167.3	3.2
1997	1113.9	0.2	397.5	1.2	131.8	35.5	199.7	1.1	203.3	3.4
1998	1025.6	0.1	359.5	1.1	109.2	26.4	226.4	1.3	169.1	3.2
1999	913.7	0.3	270.8	1.2	164.0	33.0	141.0	1.8	157.0	3.5
2000	793.2	0.2	380.0	1.4	165.4	25.6	247.9	1.7	160.0	3.7
2001	816.5	0.2	473.0	0.8	146.0	26.5	227.0	1.9	136.0	3.6
2002	1397.7	0.2	450.0	1.4	160.0	24.4	256.0	2.0	130.0	3.9
2003	1379.5	0.3	345.0	1.7	153.0	27.6	229.0	1.7	149.0	3.9
2004	1175.2	0.3	308.0	1.9	124.0	27.1	242.0	1.7	226.0	4.2
2005	1242.7	0.3	345.0	2.1	116.0	27.7	188.0	2.0	191.0	3.9
2006	1491.9	0.3	355.8	1.9	117.3	29.3	271.9	1.2	165.4	4.2
2007	1901.6	0.2	398.4	2.3	138.0	30.4	358.7	1.7	238.2	4.4
2008	1543.7	0.3	853.3	2.2	133.1	31.1	469.3	1.6	291.8	4.1
2009	1803.1	0.3	855.0	2.2	149.8	35.3	488.3	1.7	262.5	4.0

Bootstrapping—sampling with replacement—was utilized to generate frequency distributions for the annuities (Equation 2) of each land-use option. Prices and productivities were drawn from the same year to produce a sample of 1000 repetitions. By applying this procedure, we did not consider correlation between the prices and productivities from one year to the next year for the same option, but rather the correlation between prices and productivities between all land-use options. Given that time series for prices and productivity of organic banana were not available in FAOSTAT, we used, in a first attempt, the coefficient of variation of prices (65%) and productivity (22%) for conventional banana as proxies to model uncertainty for organic banana. The very important coefficient of correlation between organic and conventional banana (ρ_conv,org_) was derived from price changes of documented wholesaler prices [58, 59]. These support a coefficient of correlation of about zero between the economic returns of both variants of banana, when prices for conventional banana are on the decrease and a positive correlation, when prices for conventional banana are on the increase. Finally, we provided a coefficient of correlation of zero between economic returns for organic banana and those for other crops, similar as the correlations found between conventional banana economic return and that other crops. Given these data and assumptions, we simulated the frequency distributions of the annual economic returns for organic banana by means of Monte Carlo Simulation (MCS).

A low coefficient of correlation between economic returns of both conventional and organic banana is also supported by the finding of another author that organic price changes are actually largely independent from conventional price changes, unless changes in conventional prices are quite large [18]. However, we nevertheless tested the effect of increasing correlation between economic returns of conventional and organic banana, possibly due to—so far not observed—growing integration of both markets, by assuming ρ_conv,org_ of up to +0.7. Moreover, as our modelling led to a very high SD for organic banana with a coefficient of variation of their economic return of 81%, we also tested the impact of a lower uncertainty of organic banana on the optimal land allocation in our portfolios. Assuming lower uncertainty is well justified and may be even more realistic compared to our initial high-uncertainty scenario, because the available price data suggest that prices for organic banana are very stable, showing only 50% of the volatility compared to prices of conventional banana. A lower price uncertainty is a very important aspect, because the price uncertainty of conventional banana, which we adopt in the initial scenario to model the fluctuation of gross revenues for organic banana, dominates the large uncertainty of the economic returns. By setting the uncertainty of the crop productivity equal to zero we still observed, through the price uncertainty alone, a standard deviation of 95% compared to the combined standard deviation from crop and price volatility. Given this background information, a scenario with reduced uncertainty of economic return for organic banana appears quite realistic. In summary, we assumed in one variation of our consideration a reduction of the SD for prices from actually US$ ±55 per Mg (conventional banana) to US$ ±30 per Mg for organic banana resulting in a coefficient of variation of organic banana’s economic return of ±50%.

We faced the same challenges regarding data availability as described for organic banana with the historical data for the prices of timber. In this case, we assumed volatility in the price of timber of 10% [48]. Random prices for balsa and laurel were simulated assuming a normal distribution. The probability distributions of returns for balsa and laurel were then estimated using MCS, also with 1000 repetitions.

To calculate the expected economic returns for each of the land-use portfolios, Equation 2 was applied, while Equation 3 delivered the SD as our risk indicator for each portfolio.

## Results

### Economic return and risk for single land-use options

**Fig 2:**
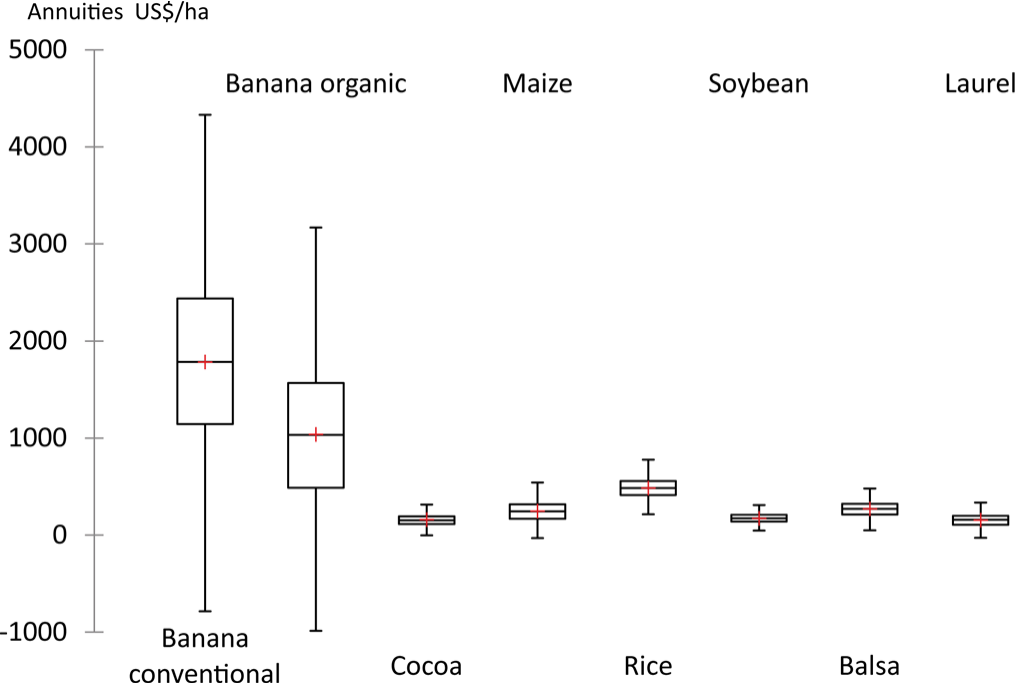
Simulated annuities for land-use options produced in the Babahoyo sub-basin. Whiskers represent the lowest and the highest annual returns in US$ ha^-1^.

**Fig 3:**
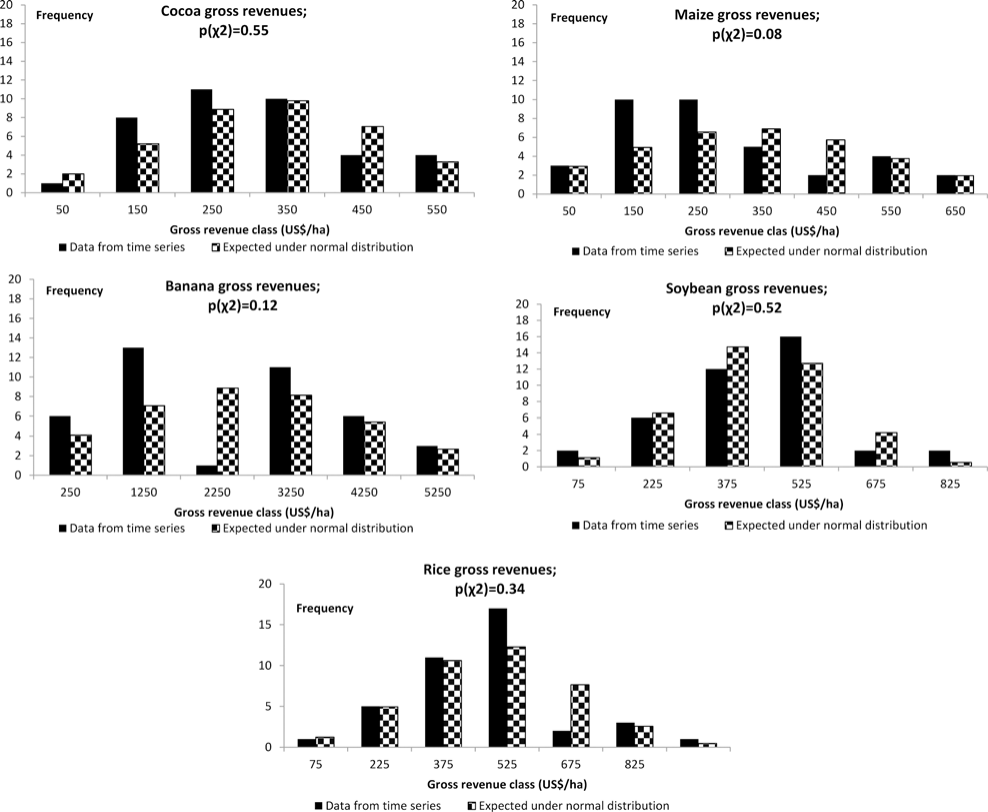
Distributions of gross revenues from time series data used for bootstrapping and expected distribution under the normality assumption. Organic bananas as well as forestry options were modelled by means of assumed normal distributions.

We will first present the simulated annual economic returns of each of the agricultural products when produced as single options (Fig. 2). Conventional banana was the option with the highest mean annual economic return (US$ 1786 ha^-1^ ±945) and also the option with the highest SD (risk). The great volatility of prices and yields which has been documented for conventional banana is the cause for these large fluctuations (Table 3, Fig. 3). For this reason, even negative economic returns are possible. Maximum calculated annual returns per ha were as high as US$ 4804, while potential annual losses were found to be as much as US$ -1557 per ha (Table 4).

**Table 4:** Descriptive statistics of financial data of land-use options considered for optimization.

Land-use option	Minimum return	Maximum return	Mean return	
	US$ ha^-1^	US$ ha^-1^	US$ ha^-1^	SD
Conventional banana	-1557	4804	1786	945
Organic banana	-1897	3913	1040	843
Cocoa	-9	473	159	70
Maize	-31	600	247	108
Rice	170	834	486	101
Soybean	-20	313	174	52
Balsa	-20	552	271	84
Laurel	-71	429	154	70

(SD: Standard deviation)

Organic banana yielded mean annual returns of US$ 1040 ±843, under this high-uncertainty scenario even with a higher coefficient of variation than conventional banana (81% versus 53% for conventional banana). Its return was substantially lower than that of conventional banana due in part to higher costs of establishment and management, but also due to a by 35% reduced productivity (see Table 1). Here, the worst case losses amounted to as much as US$ -1897.

In general, the annual economic returns for all of the non-banana options were below US$500 ha^-1^. Annual returns of rice amounted to US$ 486 ±101. An economic advantage of rice found by our modeling was that, even in the worst case, it still yielded a positive annual return—a minimum of US$ +170 ha^-1^—and thus rice may be considered the single option with the smallest economic risks. Among the annual crops, maize was the crop with the largest SD (US$ 247 ±108 ha^-1^), while soybean had the lowest SD (US$ 174 ±52 ha^-1^).

Permanent crops—forestry and cocoa—had dissimilar financial performances. For cocoa and laurel, the mean annual returns were similar—US$160 ±70 ha^-1^. Balsa however, achieved a mean economic return of US$281 ±84 ha^-1^ year^-1^. This value was higher than those for annual crops such as soybean and maize, and even in terms of risk, balsa performed better than the latter. Descriptive statistics and correlation coefficients between the land-use options are summarized in Tables 4 and 5.

**Table 5:** Correlation coefficients of land-use options.

	Banana conventional	Banana organic	Cocoa	Maize	Rice	Soybean	Balsa	Laurel
Conventional banana	1.00							
Organic banana	0.02	1.00						
Cocoa	-0.01	-0.03	1.00					
Maize	-0.06	-0.01	-0.02	1.00				
Rice	0.02	-0.03	0.43	0.02	1.00			
Soybean	0.03	-0.01	0.36	0.01	0.59	1.00		
Balsa	0.04	0.02	-0.02	0.02	-0.02	0.00	1.00	
Laurel	0.01	0.02	0.03	-0.01	-0.02	-0.02	0.08	1.00

The distribution of the gross revenues derived from time series data was largely not significantly different from an expected normal distribution (Fig. 3) with p(χ^2^) from 0.12 to 0.55. Only for maize the statistic, p(χ^2^), was with 0.08 below the required threshold of 0.10. We may thus regard the requirement for the analysis of economic portfolios for economic returns to be normally distributed as more or less fulfilled.

### Correlation between prices for conventional and organic banana

**Fig 4:**
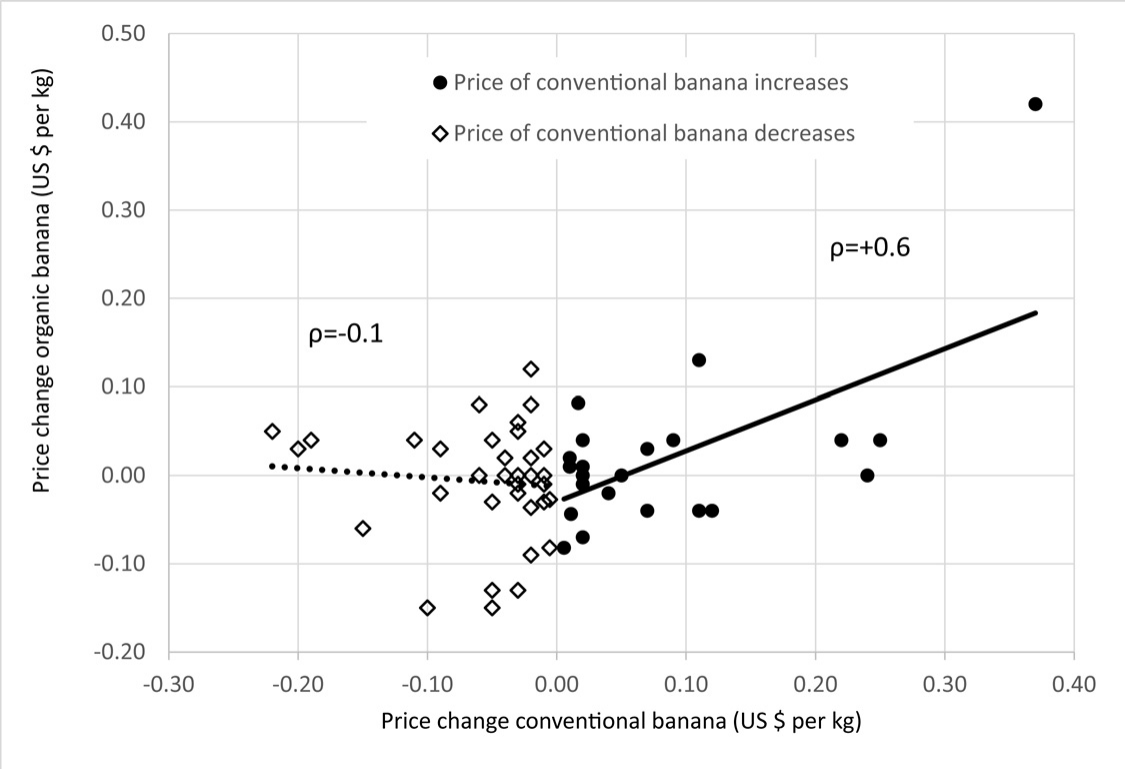
Correlation of price changes for conventional and organic banana [58, 59].

Plausible information on the correlation between economic returns is a precondition for any portfolio-theoretic analysis. The necessary data could be derived from FAO statistics for most of the crops considered (Table 5), but it is hard to be obtained in the case of organic banana. However, some time series have been documented on wholesaler prices of organic banana [58, 59]. It is due to the fact that we found that the volatility of the economic return for banana is mainly driven by price uncertainty, that the correlation between prices for organic and conventional banana may be regarded as a good indicator for the correlation between economic returns. The analysis of price changes showed that price shifts for organic banana are independent or even slightly negatively correlated with price shifts of conventional banana, when prices for conventional banana decline (ρ_conv,org_ = -0.1, see Fig. 4). However, when prices for conventional banana increase, also the prices for organic banana show a tendency to increase (ρ_conv,org_ = +0.6). This makes organic banana an ideal complement for the conventional banana, obtaining stable or even slightly increasing prices, when conventional banana price declines and when it increases, respectively.

### Forming land-use portfolios

**Fig 5:**
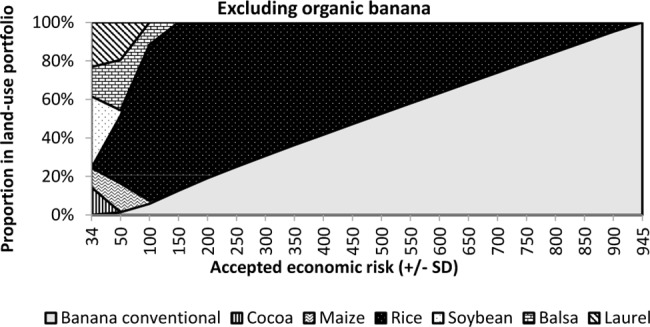
Structural composition of various land-use portfolios without organic banana for increasing levels of accepted economic risk.

In our reference scenario organic banana was not a considered option (Fig. 5). While the single option with the lowest risk (soybean) shows a SD of ±52, a diversified portfolio with 14% cocoa, 10% maize, 37% soybean, 15% balsa, and 23% laurel obtained a smaller risk of ±34, which is the minimum risk achievable for the considered land-use. However, the economic return of this portfolio is relatively small (US$ 191 ha^-1^ yr^-1^). By accepting the same level of risk as that inherent in soybean (±52) the farmer would be greatly rewarded when forming a land-use portfolio of 2% conventional banana, 15% maize, 38% rice, 27% balsa, and 18% laurel with an annual expected economic return of US$ 352 ha^-1^ yr^-1^. This is ≈US$ 160 more than achievable at the risk minimum and the risk to be tolerated is still not higher than the risk of single soybean.

Tolerating more risk results in higher expected economic return in our example, which is normal—at least when starting from the portfolio with the minimum of risk. Our land-use portfolios are highly diversified for farmers with low risk tolerance and contain forestry options as well, while the proportion of high-return conventional banana increases with increasing risk tolerance (Fig. 5). However, rice is also included over a large range of possible risk tolerances to diversify risks, while only those farmers who would totally disregard risks should work with conventional banana as a stand-alone option.

**Fig 6:**
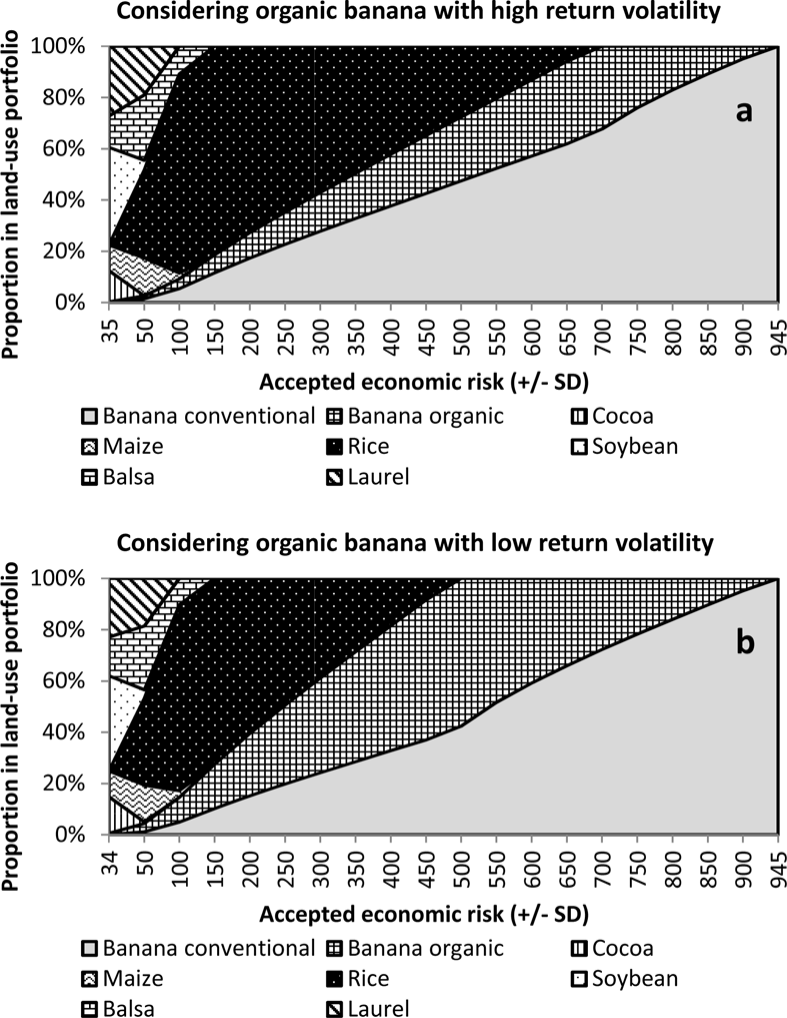
Structural composition of various land-use portfolios for increasing levels of accepted economic risk when organic banana is included and has high (a) or low economic risks (b).

Interestingly, when considering organic banana as an option available for tropical banana farmers in Ecuador, this option would be included in the risk-return-efficient portfolios for a very large range of tolerated risks, and this despite its, in this initial high-uncertainty scenario, quite large own risk as a single option. This means that organic banana increases the expected economic return compared to portfolios excluding this crop. The magnitude of this effect will be demonstrated later. Organic banana obtains proportions between 1%, for a low tolerated risk of ±50, and 32%, for a tolerated risk of ±650 (68% of the SD of pure conventional banana). The proportion of organic banana sinks again to 5% for a very high tolerated risk level of ±900 (Fig. 6a).

As organic banana holds a quite high simulated risk as a single option in the initial scenario, rice plays a major role in the portfolios containing organic banana to hedge against the uncertainties involved with the organic crop. Embedded in a portfolio with rice and conventional banana, the same economic return as with pure organic banana (US$ 1040 ha^-1^ yr^-1^) may be achieved by a diversified land-use portfolio, but at a risk of only ±369 instead ±843 for pure organic banana. Here, the portfolio structure would be 35% conventional banana, 19% organic banana, and 46% rice. This diversified land-use portfolio would thus hedge the great simulated risks of pure organic banana quite effectively.

However, the modelling results depend strongly on the assumptions made: a) If the simulated very high risk of organic banana was not ±843 (coefficient of variation ≈80%), but ±506 (coefficient of variation ≈50%), the portfolio’s structure changed significantly. Reduced price volatility for organic banana is not unrealistic according to their much lower observed historical price changes compared to conventional banana. If we leave one outlier aside we find price changes between-0.23 and +0.26 US$ per kg for conventional and changes between-0.15 and +0.12 US$ per kg for organic banana (Fig. 4). Acknowledging a lower price risk, the proportion of organic banana was greatly increased, up to 57%, while the proportion of rice was reduced (Fig. 6b).

b) If we assume an increased coefficient of correlation between the economic returns of organic and conventional banana (ρ_conv,org_ of +0.5 or +0.7), then the sensitivity of the results largely depends on the assumed price risk of producing organic bananas. For the case that the risk of organic banana is as high as modelled in our initial scenario, the increased correlation would reduce the proportion of organic banana to a maximum of only 1% (ρ_conv,org_ of +0.5). Organic banana is then replaced by rice. However, if a reduced price risk is considered, which appears to be a quite realistic assumption, the proportions of organic banana remain relatively stable, even if the correlation, ρ_conv,org_, of the economic returns is quite high (ρ_conv,org_ +0.5 or +0.7). For example, given ρ_conv,org_ = +0.7, organic bananas still hold a proportion of 8% for a very high tolerated SD of ±900.

**Fig 7:**
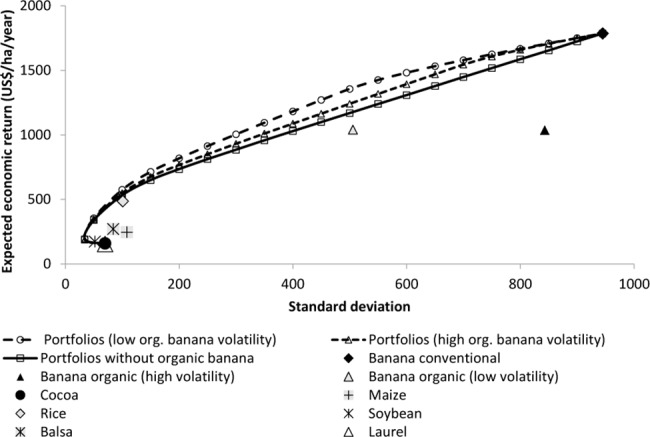
Maximum expected economic return achievable of diversified land-use portfolios for various levels of potentially accepted economic risk compared to economic returns of single land-use options.

Finally, through including organic banana into their land-use portfolios, farmers may increase their economic returns for a large range of tolerated economic risks (Fig. 7). Farmers may obtain 7% higher economic return (+US$ 96 ha^-1^ yr^-1^) from a land-use portfolio including an assumed high risk organic banana, when tolerating an economic risk of ±700. If a reduced price risk of organic banana is acknowledged, farmers could even obtain US$ 187 ha^-1^ yr^-1^ more, when accepting an economic risk of ±500 and including 57% organic banana. In summary, although organic banana appears less attractive as a single option, this option may, when embedded in land-use portfolios together with other crops, improve the economic return of Ecuadorian banana farms. We have, thus, found supporting evidence through our modelling approach for our hypothesis: H: “The inclusion of organic banana into efficient economic land-use portfolios in Ecuador is driven by the uncertainty of their economic return”

## Discussion and Conclusions

### Comparing conventional and organic agriculture

Long-term sustainability of agriculture will hardly be attainable if current, conventional intensive practices continue to be applied. Agricultural intensification must therefore be coupled with sustainable land-use practices in order to be efficient [25, 60]. Nevertheless, the economic assessment of such a change towards more sustainable ways of producing food must receive more attention, if we are to better understand the decision-making process of resource allocation at the farm level as well as at the landscape and national levels [22, 23].

We have assessed the effects on the overall economic returns of a farm by considering organic banana and forestry options as potential land-use practices for future land-use portfolios and found this perspective more informative than the existing approaches where conventional and organic productions systems are seen as being mutually exclusive [13, 45, 46]. In the region under study, intensive and very high yielding agriculture (banana) is the *business as usual* alternative. Due to high capital requirements of the *business as usual* banana option, we do not assume in our modelling that the banana farmers have limited access to capital.

The economic returns for both conventional and organic banana were impressive and very high in comparison with the other crops. Wunder has already shown that banana achieved by far the highest gross incomes in Ecuador, even when compared with other high profit crops such as sugar cane, potatoes, or African palm [61]. Another study [62] has reported high annual economic return for banana between ~US$ 1200 to 2000 per ha for India, but lower annual economic return has been documented for banana in Bangladesh (~US$ 870 per ha). However, the estimates for economic return of bananas reported in the literature are extremely variable, with annual economic returns up to ~US$ 3800 per ha, a value being reported for Bangladesh [63], while the maximum included in our Monte Carlo simulations was US$ 4808 per ha for conventional banana. These studies show that the high computed annual economic returns for banana (i.e. averages of US$ 1786 per ha for conventional and US$ 1040 per ha for organic banana) in our study range in a realistic order of magnitude.

While the forestry options diversified the land-use portfolios effectively rather for very cautious, risk-avoiding farmers, organic (and also conventional) banana enter the land-use portfolios only, if higher risks are tolerated. Regarding organic banana, we found that despite the possibly too high simulated risk it is well be balanced in land-use portfolios containing rice and conventional banana, if the correlation between economic returns of organic and conventional banana is not too high.

Nevertheless, the degree of diversification was limited when the combination of land-use practices included high-yielding crops as conventional banana. In our case, including high-yield banana as a portfolio option certainly lowered the resulting degree of land-use diversification, limiting the portfolio often to only a few land-use options. But still, in every portfolio we generated (except the maximum risk portfolio), we had at least two crops, with no single-crop turning out to be optimal.

One potential criticism to our model could be that only a modest ecological benefit can be expected because a high degree of diversification was not achieved—unless we assumed great risk aversion. Nevertheless, the fact that a land-use portfolio consists of only few options does not necessarily mean that a similar landscape structure to those observed in monocultures will be reproduced. Growing crops in relatively small compartments is one way to break up the landscape and also to achieve reduced erosion caused by wind and water, while still allowing for some level of mechanization [25]. Additionally, structural elements such as hedgerows should be implemented on around 5% of the land in order to enhance the structural diversity of the landscape. However, including these areas might represent a reduction in the amount of land available for farming and thus result in an accordant reduction in revenue. The considerable future challenges in economic comparisons of organic and conventional agriculture include the quantification of more synergies or antagonisms. While we found rather synergistic risk interactions of conventional and organic banana farming, also the interaction between pest management in conventional parcels and the susceptibility for pests in organic parcels should be further investigated.

Reductions in economic returns by means of pests might also be the main constraint to the implementation of sustainable agricultural practices. We assumed a reduction in productivity of 35% for organic compared with conventional banana. This reduction is similar to the yield losses which organic plantations may face compared with conventional plantations, due to infestation with the Black Sigatoka fungus (*Mycosphaerella fijiensis*) [45]. Still, more and better information can perhaps encourage farmers to adopt practices leading to environmental improvements. This is particularly true when changes in farming and land-management practices intended to enhance ecosystem services also benefit farmers themselves. In situations when such changes imply a reduction in farmers’ income, implementation can only be achieved through enforced regulations or when some form of compensation is provided [16].

### Attractiveness of diversification and possible impacts of option values

Diversification is an acknowledged strategy for coping with risks; however, if farmers have access to other means of hedging risks, the effects might be undermined [24]. Based on the principle of risk-return reciprocity, the planting of high-yield crops corresponds to higher risk [64], as confirmed by our study. Wealthy farmers, consequently, not only hold portfolios which require higher levels of investment, they are also disposed to receive higher average profits per unit of wealth despite their greater exposure to risk [65]. As farmers become wealthier they may tend to be less averse to risk and also tend to be less interested in any form of risk-reducing intervention [23]. Although our results have shown that some diversification is highly meaningful, even for less risk-averse farmers, more intensive diversification is probably more important for poorer farmers [65]. Poorer farmers are both more exposed to and more averse to risk, and they usually lack strategies to hedge against risks [16]. Ultimately, wealthier farmers can afford better technologies and infrastructure and have better access to information [23].

One can also speculate about how options inherent in a flexible conversion strategy could alter the structural composition of the land-use portfolios obtained. For example, farmers could speculate for the optimal timing for conversion to organic banana, when particularly high prices are to be expected for this crop. A similar question has been investigated for the field of forest science, where Knoke and Wurm have adopted the Monte Carlo simulation technique to test the consequences of a flexible timing of timber harvest against a more conservative strategy with pre-defined harvest times [39]. The flexible harvest strategy allowed timber harvesting only, when a before defined reservation price was exceeded by simulated timber price scenarios. This strategy led to higher average timber prices, but also to variable additional costs for holding timber capital on the forest land by postponing the harvest times. While the average net present value could be enhanced by the flexibility strategy, its SD showed the tendency to increase, too. Although the variation of the timber prices achieved was reduced through the flexibility strategy, the then variable harvesting times increased the variability of the simulated net present values. In a mixed forest the flexible harvest strategy, thus, led to an increase of the proportions of the less risky (but also less profitable) timber species. This underlines the importance of the economic uncertainty of the single land-use options for the composition of land-use portfolios. If we, for example, could increase the economic return for organic banana by utilizing flexible management options and would increase the economic uncertainty at the same time, a reduced proportion of organic banana in the land-use portfolios could be the result.

### Are organic farming and forestry appealing to relatively wealthy farmers?

The alternative we explored was the introduction of organic farming on part of the farms, as a strategy to enhance ecosystem services provision while also reducing health hazards caused by the application of agrochemicals and reduce the dependency of farmers to rising prices of fossil fuels [15]. Producing organic crops provides an opportunity for farmers in developing countries to participate in new markets [16]. Nevertheless, a shift towards organic production is tricky, and also quite risky, due to the changes and uncertainties which occur during the transition. Yield decline may be an important obstacle for farmers who are used to producing high-yielding crops like banana. However, for such a situation our study proved great advantages of embedding the organic banana parcels in a more diversified portfolio together with other land-use practices. The effect of transition on farmer’s revenues can be better managed, provided that the trend of the price premium for organic products remains stable and the market is still growing without strong integration between the markets for organic and conventional products [18]. However, the certification process should be adapted to the conversion towards organic products only on parts of the farms, which is not accepted by all certification bodies [15]. Thus, increased flexibility during the certification process and permanent support is essential to enable farmers to move from conventional to organic farming [53].

Moving now to the forestry options, we were surprised that in the portfolio calculated for very risk-averse farmers, the options including trees accounted for about 40%. Even though forestry is a non-traditional land use in the area where this research took place, it has great potential as a complement to agriculture, especially when implemented using short-rotation species. Afforestation is particularly valuable when used to restore abandoned farmlands [66]. Reducing the life span of forestry options may have a tremendous impact on farmers′ investment decisions, because one of the primary obstacles to investing in forestry is the long-term nature of most forestry projects, which makes farmers reluctant to invest in plantations [42]. Species such as balsa, which is able to deliver returns after only five years, might completely change the perception of investors. This factor is especially important in the tropics, where the lack of financial incentives for investing in forestry activities sets the scene. We believe that the potential of forestry could be increased even more if accompanied by appropriate measures.

### Policies to encourage adoption of sound practices

As a final point, we insist that implementation of sustainable practices in agriculture will only be possible if accompanied by appropriate scientific advice, policies and supported by fair markets [5]. Farmers will not automatically shift to this type of agriculture, as the economic returns from conventional agriculture are still higher. Up to now, incentives to foster sustainable land-use practices are insufficient to induce socially desired levels of adoption [1].

The role of governments and development agencies in the coming years is that of supporting farmers in implementing sustainable, possibly organic practices by means of technology transfer and capacity-building. We must keep in mind that applying sound practices requires time and expertise, and farmers need training [15]. In addition, governments should contemplate policies that will facilitate this transition by means of financial incentives, tax reductions and access to certification bodies that help regulate organic agriculture and sustainable forestry [15]. Finally, given that the development and application of technologies for sustainable farming is expensive [4], a strong public role will continue to be necessary to support research and diffusion of knowledge among farmers, especially poorer ones [67–69].

## Supporting Information

S1 Dataset Economic coefficients and results of Monte-Carlo simulations. (XLSX)
